# Radioactive contamination transported to Western Europe with Saharan dust

**DOI:** 10.1126/sciadv.adr9192

**Published:** 2025-01-31

**Authors:** Yangjunjie Xu-Yang, Charlotte Skonieczny, Sophie Ayrault, Jean-Sébastien Barbier, Rémi Bizeul, Octave Bryskere, Pierre-Alexis Chaboche, Thomas Chalaux-Clergue, José A. Corcho-Alvarado, Anthony Foucher, Alice Karsenti, Maxime Leblanc, Germán Orizaola, Amélie Plautre, Stefan Röllin, Nirina Taraconat, Nicolas Tenaud, Ana Elisa Valdés, François Dulac, Olivier Evrard

**Affiliations:** ^1^ Laboratoire des Sciences du Climat et de l'Environnement (LSCE/IPSL), Unité Mixte de Recherche 8212(CEA-CNRS-UVSQ), Université Paris-Saclay, 91191, Gif-sur-Yvette, France.; ^2^ Université Paris-Saclay, CNRS, GEOPS, 91405, Orsay, France.; ^3^ Université Clermont Auvergne, CNRS, Institut de Chimie de Clermont-Ferrand, 63000 Clermont-Ferrand, France.; ^4^ International Research Fellow of Japan Society for the Promotion of Science, 102-0083, Tokyo, Japan.; ^5^ Institute of Environmental Radioactivity, Fukushima University, Fukushima City, 960-1296, Japan.; ^6^ Spiez Laboratory, Federal Office for Civil Protection, 3700 Spiez, Switzerland.; ^7^ IMIB-Biodiversity Research Institute (Univ. Oviedo-CSIC-Princip. Asturias), 33600 Mieres-Asturias, Spain.; ^8^ Zoology Unit, Department of Biology of Organisms and Systems, University of Oviedo, 33071 Oviedo, Asturias, Spain.; ^9^ Université Paris Cité, Institut de physique du globe de Paris, CNRS, 75005, Paris, France.

## Abstract

The Reggane region, where the first French atmospheric nuclear tests were conducted in the 1960s in Southern Algeria, is located in one of the most active dust source regions responsible for recurrent massive Saharan dust events reaching Western Europe and affecting air quality. After a major outbreak in March 2022, a citizen participative science campaign was launched to study the radioactivity born by the dust. One hundred ten deposit samples were collected from six countries in Western Europe with 53 demonstrated as scientifically representative. Geochemical and mineralogical sample analyses combined with satellite observations and back trajectory calculations confirmed an origin from South Algeria, including the Reggane site. Plutonium isotopic signatures, a unique nuclear bomb fingerprint, remained in the range of the global fallout signatures largely dominated by US and former USSR nuclear tests, significantly different from French fallout signatures. Radioactive contamination detected in all samples did not, however, present a risk to public health in terms of radioactivity exposure.

## Teaser

Artificial radioactivity transported by Saharan dust to Western Europe is from global fallout and has a negligible health effect.

## INTRODUCTION

Dust plays an important role in the Earth’s climatic system (*1*). These mineral particles can affect radiation, modify cloud optical properties and lifetimes, as well as affect precipitation processes (*2*, *3*). Once deposited, mineral dust also provides nutrients for terrestrial plant development (*4*) as well as for phytoplankton (*5*), having impacts on global CO_2_ sinks (*5*). Mineral dust can also affect air quality and public health (*6*). Notably, chemical composition and particle size distribution are critical when considering the potential impacts of mineral dust on human health (*7*). Desert dust particles in suspension in the atmosphere are emitted from regions with easily erodible and dry soils, swept by strong surface winds (*2*). The Sahara Desert and the Sahel region in North Africa supply 50 to 70% of global dust sources (*8*). Each year, 400 to 2200 Tg of Saharan dust is emitted by the North African region (*9*), with 12% transported toward Europe (*10*), where most of the dust is deposited during sporadic events with high dust load (*11*). These events are often associated with so-called atmospheric rivers, carrying tropical air rich in water vapor, clouds, and desert dust from North Africa to Europe (*12*). In Southwestern Europe, these events occur generally in early spring (*13*). Particulate air quality in Europe is often heavily affected during these dust events (*14*). In addition to the general pollution issue, the potential transport of radioactivity associated with Saharan dust that may originate from French nuclear tests conducted in the 1960s in Southern Algeria remains a major matter of concern among the population and has been widely debated in the scientific community (*15*–*17*). Only a few studies of Saharan dust events, generally collected at individual air quality measurement stations along the Mediterranean Sea (*15*) or modeled (*16*), were conducted and investigated multiple dust properties including artificial radioactivity. Regarding radionuclide concentrations in dust, previous studies concluded that these dust events only contributed to the long-term background level of artificial radionuclides remaining at trace levels in the atmosphere worldwide (*15*, *16*). Despite these previous results, some news, media, and social networks exclusively attributed the radioactivity borne by this dust to French nuclear weapon tests conducted in the early 1960s in Southern Algeria (*18*–*20*), and they suggested that the dust radioactivity levels may be significant, increasing the general public concerns associated with these recurrent dust events.

During the winters of 2020–2024, several intense North African dust intrusions affected Europe with durations and intensities that had never been recorded before (*21*). A particularly massive dust outbreak occurred in March 2022. This dust event, which started on 15 March, was exceptional dust transport from North Africa to the Western Mediterranean region and broke all records in terms of duration and intensity, notably affecting air quality in Western and Central Europe (*21*–*23*). On the basis of the analysis of satellite images, South Algeria was identified as the main dust source of this dust outbreak (*21*). This part of Algeria includes areas where the “Reggane” series, a group of four atmospheric atomic bomb nuclear tests, was conducted by France between February 1960 and April 1961 (*24*). The estimated total power of these four tests is 72.7 Kt, 0.02% of the total nuclear weapons test power released worldwide between 1945 and 1980 (*25*).

**Fig. 1.**
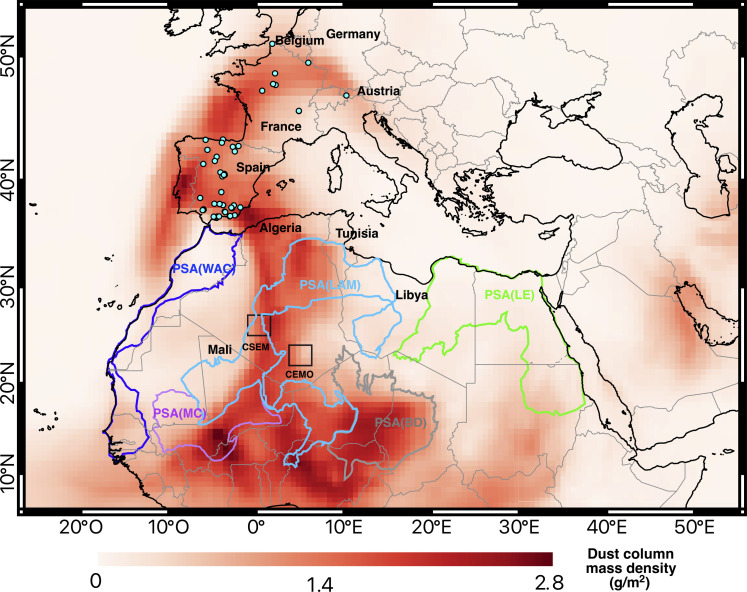
March 2022 dust event sampling sites and dust column mass density (grams per square meter). Blue points represent the 53 scientifically representative samples of the Saharan dust collected during the science citizen campaign (see detailed description in the Supplementary Materials).The color scale corresponds to the estimated dust concentration averaged for the 2022-03-15 00Z to 2022-03-16 23Z temporal window based on satellite measurements and MERRA-2 model from the Giovanni NASA Earth data base (https://giovanni.gsfc.nasa.gov/giovanni/). The CSEM and the CEMO where nuclear tests were conducted are marked by squares. The five PSAs as defined by Guinoiseau *et al.* (*30*) based on geochemical fingerprint studies and geological subdivision of North Africa are marked by colored areas. PSA(LAM) corresponding to Libya-Algeria-Mali, PSA(WAC) corresponding to West Africa Coast, PSA(MC) corresponding to Mali Center, PSA(BD) corresponding to Bodélé Dépression, and PSA(LE) corresponding to Libya-Egypt.

Given the significance of the event, we initiated on 17 March 2022 a citizen participative science campaign with the help of X (formerly known as Twitter) social media (*26*) to examine the potential transport of radioactive isotopes such as cesium-137 [^137^Cs, half-time (*t*_1/2_) = 30.2 years], plutonium-239 (^239^Pu, *t*_1/2_ = 24,110 years), and plutonium-240 (^240^Pu, *t*_1/2_ = 6563 years) in association with Saharan dust. Thanks to this initiative, 110 samples of Saharan dust deposits were collected by 69 citizens in six Western European countries (Austria, Belgium, France, Germany, Luxembourg, and Spain), at latitudes ranging between 37° and 51°N (see Materials and Methods). We combined grain size distribution, clay mineralogy composition, and various geochemical analyses [elemental concentrations and lead (Pb) isotopes], radionuclide contents (^137^Cs, ^239+240^Pu), and signatures (^240^Pu/^239^Pu) in the collected dust. We also established a general contextualization of the event using satellite products and airmass back trajectories. This multiproxy approach allowed us to first select the scientifically representative samples (53 of 110) collected by the citizen science experience, mainly based on particle size distribution and sampling date (see the Supplementary Materials) (Fig. 1), and then to precisely trace back the provenance of the dust from across the very large North-West African region and to determine their radioactivity contents and source. The current study represents one of the first multiproxy datasets, including detailed artificial radionuclide analyses in a large set of atmospheric deposits collected at multiple locations across Western Europe during a single widespread dust outbreak event, allowing us to directly address the question associating recurrent Saharan dust events and their potential radioactivity contamination supply to Western Europe.

## RESULTS AND DISCUSSION

### Southern Algeria’s atmospheric nuclear weapon test region as a main dust source area

**Fig. 2.**
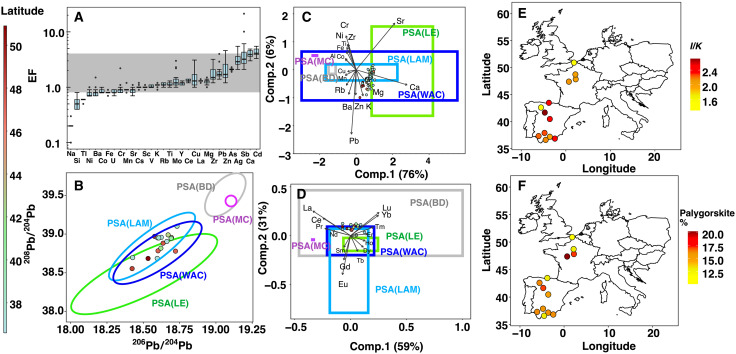
Geochemical and clay mineralogical characterization of the Saharan dust collected by the citizen science experience. In the different panels, dots symbolized the dust samples collected during the March 2022 event. (**A**) EFs of all analyzed elements (except REE heavier than Ce) ranked by the mean value. The gray area represents the range between 0.8 and 4, which indicates a predominant crustal origin (*28*). (**B**) ^208^Pb/^204^Pb versus ^206^Pb/^204^Pb results showing that the lead isotopes signature of a subset of scientifically representative samples are constrained in PSA(LAM). (**C**) Compositional biplot of XRF data. Comp.2, Component 2. (**D**) Compositional biplot of REE. For (B) to (D), the scale of colors of the sample’s dots correspond to their latitudes of collect (on left, blue to red color scale); the signatures of North African PSA (*30*) are represented by colored ellipses or frames with corresponding legend. (**E**) Illite/Kaolinite (*I*/*K*) clay mineralogical ratio and (**F**) palygorskite clay mineral percentages results plotted on a map of Europe, with scale color (on right, yellow to dark red) representing the *I*/*K* ratio and palygorskite%.

**Fig. 3.**
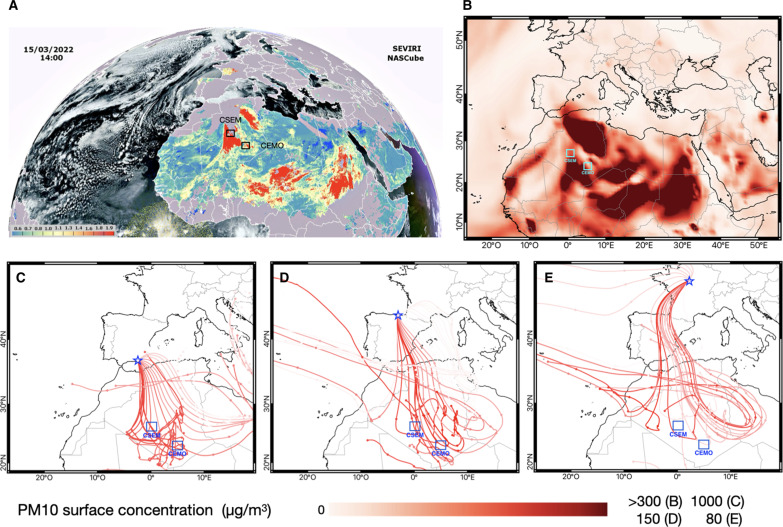
March 2022 dust event satellite and back trajectories analyses. (**A**) Sensing images of aerosols optical depth measured by NASCube at 14:00 p.m. the 15 of March 2022. (**B**) Surface PM_10_ concentration (micrograms per cubic meter) for the 15th March 2022 derived from CAMS reanalysis of atmospheric composition (*41*). Bottom (**C** to **E**) HYSPLIT series of backward trajectories arriving at 3000-m height in South (Almeria, 37°N) and North (Alceda, 43°N) of Spain and Central France (Sigloy, 48°N) with one trajectory calculated every 3 hours between 15 and 18 March 2022. The trajectory length is 6 days with a dot every 24 hours. The color of the trajectories corresponds to the PM_10_ levels (micrograms per cubic meter) measured at the ground level by monitoring site near the sampling site at the arriving time of trajectories, and dark red colored trajectories are responsible for the PM_10_ concentration peaks measured at the sampling site between 15 and 18 March 2022.

Elemental enrichment factors (EFs), a widely used metric for determining how much the presence of an element in a sample medium has been increased by human activities relative to the Upper Crust Composition (UCC) (*27*), of dust samples collected during the citizen science experience generally ranged from 0.8 to 4 (Fig. 2A), confirming a crustal origin of the deposits (*28*). The relatively high calcium (Ca) enrichment (Fig. 2A) is consistent with the common high Ca content of Saharan dust originating from North Africa that can be three to four times higher than that found in the UCC because of the regional geology dominated by limestones and carbonate-rich soils (*29*–*31*). Compositional biplot of half-quantitative x-ray fluorescence (XRF) analysis indicates that elemental ratios coincided with the ranges found in three North Africa potential source areas (PSAs; Libya-Egypt, Libya-Algeria-Mali, and West Africa Coast; Fig. 2C), as determined by other studies relying on geochemical fingerprints and satellite observations (*30*, *32*). The systematic occurrence of palygorskite clay mineral in the samples (between 10 and 20% in abundance; Fig. 2F) suggests a North African provenance, more specifically from the anti-Atlas Morocco region, Algeria, or Tunisia (*31*). Lead (Pb) isotope analyses more precisely attribute the origin of the dust to the Libya-Algeria-Mali PSA (Fig. 2B) (*30*, *33*–*36*). The Libya-Algeria-Mali PSA is the second largest dust source on the African continent, following the well-known Bodélé Depression (*37*). This major source provides ~13% of the North African annual dust emissions (*38*) and supplies most of the dust transported to the Mediterranean Sea and Europe (*21*, *39*). Mineralogical and geochemical signatures of the dust investigated here are consistent with the North African Sandstorm Survey (NASCube) (*40*) images, which detected intense and large-scale sandstorms in Southern Algeria, Northern Mali, and Western Libya during the dust event (Fig. 3A). As previously noted, this Libya-Algeria-Mali PSA encompasses the Reggane region where the first four French atmospheric nuclear tests were conducted at the Centre Saharien d’Expérimentations Militaires (CSEM) as well as regions near the Tamanrasset where 13 underground nuclear tests were conducted at the Centre d’Expérimentations Militaires des Oasis (CEMO) in the 1960s [Fig. 1; International Atomic Energy Agency (IAEA), 2005]. The Reggane region represented one of the most active dust sources in North Africa during the March 2022 outbreak, especially during daytime on 15 March 2022 (Fig. 3A). The rare-earth element (REE) compositional variation of the samples corresponds to a limited range of values covered by the Libya-Algeria-Mali PSA (Fig. 2D), suggesting a relatively constrained region of provenance within the Libya-Algeria-Mali PSA. The clay mineralogical illite over kaolinite (*I*/*K*) ratio varied between 1.3 and 2.7, with a mean value of 2.2 (Fig. 2E), which is also consistent with the characteristics of Algerian surface sediments (*31*). Backward trajectories calculated between 15 and 18 March 2022 for three latitudinally different localities from Southern Spain to Central France (40°, 42°, and 46°N) (Fig. 3, C to E) all indicate Southern Algeria as a major dust provenance area swept by surface winds, before crossing the Mediterranean Sea and reaching these localities. According to the European Environment Agency, concentrations in particulate matter with size lower than 10 μm (PM_10_) reached particularly high values during the event [~1100 μg m^−3^ in southern Spain (~40°N); ~700 μg.m^−3^ in central Spain (~42°N), and ~150 μg.m^−3^ in central France (~46°N)]. The backward trajectories coincided well with the high levels of PM_10_ detected in Western Europe at the ground level and were associated with the intense sandstorms detected 1 to 2 days before in Southern Algeria by NASCube. This is also consistent with the Copernicus Atmosphere Monitoring Service (CAMS) reanalysis of atmospheric composition (*41*) showing high levels of surface PM_10_ concentrations in Southern Algeria (Fig. 3B and fig.S3). In conclusion, the mineralogical and geochemical signatures of the March 2022 Saharan dust samples combined with the associated analyses of backward trajectories, NASCube aerosol optical depths, and CAMS surface dust PM_10_ concentrations all robustly confirm that dust deposited over Western Europe mainly originated from the Southern Algeria region, encompassing the Reggane site where atmospheric nuclear tests were conducted by France in the 1960s.

### Saharan dust ranges within the global fallout artificial radioisotope signature

**Fig. 4.**
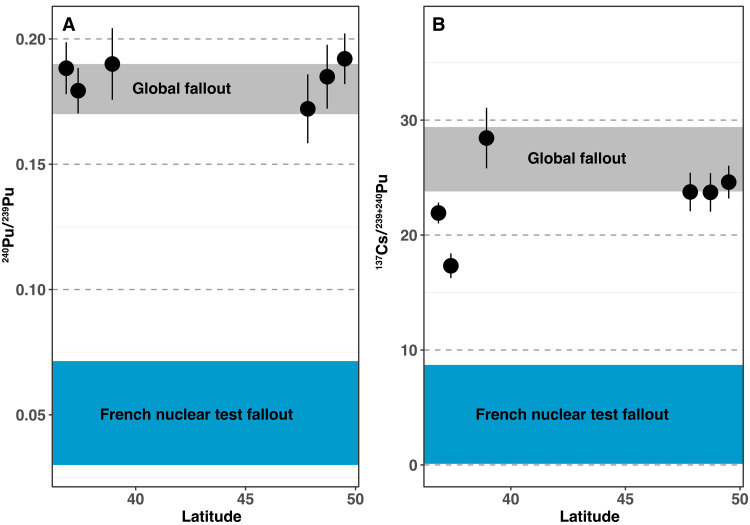
March 2022 Saharan dust samples radioisotope signature. (**A**) ^240^Pu/^239^Pu and (**B**) ^137^Cs/^239+240^Pu values of Saharan dust collected during the 2022 episode along with the signatures of potential sources (*24*, *42*–*46*).

Plutonium isotopic ratios of fallout associated with French nuclear tests conducted in Algeria are expected to be lower than 0.07 due to different fuel compositions, explosion altitudes, and detonation systems compared to US and Union of Soviet Socialist Republics (USSR) tests (*42*–*47*). Here, ^240^Pu/^239^Pu ratios measured in samples of the March 2022 Saharan dust event ranged between 0.172 ± 0.014 and 0.192 ± 0.010 (Fig. 4A), with a median value of 0.187 ± 0.016. These ratios are comparable to those measured in the PM_10_ dust particles collected during a 2004 dust event in France, with a mean value of 0.194 ± 0.033 (*15*). The ^240^Pu/^239^Pu ratios measured in samples of March 2022, therefore, coincide with the mean ratio of 0.18 ± 0.01 reported for the global fallout signature (Fig. 4A), largely dominated by US and former USSR nuclear tests and which tagged soils from around the world (*42*, *43*). This global origin of radionuclide in the dust is confirmed by the ^137^Cs/^239+240^Pu activity ratio, ranging from 17.3 ± 1.1 to 28.4 ± 2.6, which also fall within the range of the global fallout [~23.8 to 29.4, decay-corrected to 2022 (*44*); Fig. 4B]. These signatures are significantly different from the ^137^Cs/^239+240^Pu activity ratio attributed to the French nuclear tests conducted in Algeria with values estimated to 0.1 to 8.7 for atmospheric nuclear tests conducted in Reggane (decay-corrected to 2022; IAEA, 2005). In conclusion, radionuclide signatures detected in Saharan dust collected in 2022 remained in the range of the global fallout found as a background signal in soils worldwide, and they significantly differed from the characteristics of the French atmospheric nuclear tests conducted in Southern Algeria.

### Negligible health effect of radioactive contamination of Western Europe by Saharan dust

**Fig. 5.**
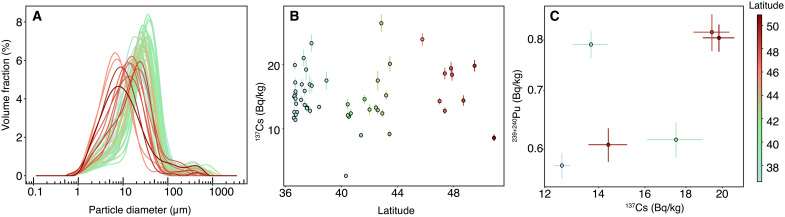
March 2022 Saharan dust sample particles size and radionuclides content. (**A**) Grain size distribution and (**B**) ^137^Cs activities of the 53 scientifically representative Saharan dust samples. (**C**) ^239+240^Pu activities as a function of ^137^Cs activities of selected samples. The color is indicative of sample location’s latitude.

Cesium radionuclide (^137^Cs) activities in the collected Saharan dust varied between 2.7 ± 0.3 and 26.4 ± 1.4 Bq kg^−1^ (Fig. 5B), with a median value of 14.4 ± 0.8 Bq kg^−1^. These values are consistent with that found in dust deposited on snow in the Pyrenees, France, during a dust event in 2021, with ^137^Cs radionuclide mass activities of 19.0 ± 0.5 Bq kg^−1^ (*48*). It is, however, lower than those measured in finer suspended particles (PM_10_) during a dust event in 2004 in France (ranging between 32 and 170 Bq kg^−1^) (*15*). March 2022 Saharan dust deposit grain size modes decreased with increasing latitudes (Fig. 5A) due to gravitational settling (*2*) and ranged from 7 to 37 μm, remaining within the silt size class. ^137^Cs being enriched in the finest clay particles (*49*), the difference in cesium activities with the 2004 PM_10_ dust samples event can be attributed to this grain size effect. ^239+240^Pu activities in the Saharan dust samples varied between 0.57 ± 0.02 and 0.82 ± 0.04 Bq kg^−1^. These activities are comparable to those reported in dust deposits from Saharan events that occurred in February 2004, May 2004, July 2005, and May 2008 (*15*, *50*).

The positive correlation between ^239+240^Pu and ^137^Cs radionuclide mass activities (Fig. 5C) suggests that both radionuclides share the same origin (*44*, *51*). As a result, ^137^Cs found in the Saharan dust deposited across Europe during the March 2022 episode is also attributed to the global fallout rather than to the nuclear tests conducted by France in Algeria, as indicated by ^240^Pu/^239^Pu atomic ratio (Fig. 4A). Even if supplying some artificial contamination, Saharan dust ^137^Cs contents are much lower than the threshold value authorized by the European Union (EU) in food products [1000 Bq kg^−1^ for cereals, vegetables, fruit, meat, milk, and dairy products and 400 Bq kg^−1^ for infant food as examples (*52*)]. Furthermore, in the case of intense Saharan dust outbreaks in Europe, the specific activities of ^137^Cs in air ranged around 1 to 7 μBq m^−3^ in suspended PM_10_ particles (*15*). Supposing that the volume of air consumed by a person in 1 day is 11 m^3^ (*53*), the inhalation dose due to the ^137^Cs exposure in breathed air amounts to 11 to 77 μBq day^−1^, which equals to 77 to 540.10^−12^ mSv day^−1^ [inhalation dose conversion factor (7.10^−9^ Sv Bq^−1^)] (*54*). These values are 10^8^ times lower than levels authorized by EU (*55*) (3.10^−3^ mSv day^−1^). We thus conclude that the health effect of radioactive contamination of Western Europe by Saharan dust is negligible.

Overall, from this unprecedented multiparticipants and multiproxy study, we conclude that major Saharan dust supplies to Western Europe, even if impressive, recurrent, and sweeping large areas including those where nuclear tests were done in the past do not present risk for public health in terms of exposition to artificial radioactivity. As for other topics in Geosciences [e.g., FRIPON “Fireball Recovery and InterPlanetary Observation Network (fripon.org)” (*56*)], we confirm with this study that participative science can contribute to investigating challenging environmental questions with a spatial coverage of sampling that would have been unachievable by individual researchers. In the future, a dedicated participative network could be created to gather, better prepare (e.g., better training in clean collection protocol), and alert citizens interested in participating solving these types of environmental scientific questions. It will help maximize the samples’ scientific representativity and contribute to a better transfer of scientific knowledge to the general population.

## MATERIALS AND METHODS

### Citizen science dust sampling campaign

A few days after the beginning of the Saharan dust storm, a call for citizen participation was launched on X (formerly known as Twitter) (https://x.com/EvrardOlivier/status/1504342715932282883). The active participation of the public allowed us to gather 110 samples collected in six different European countries including France (14 samples), Austria (12 samples), Spain (80 samples), Belgium, Luxembourg, and Germany (1 sample in each of these three countries) (fig. S1). All samples consisted of dry powder and were collected more or less shortly after deposition, during a period ranging between 13 March and 23 April 2022, and on variable types of exposed surfaces (e.g., metal roof, snow, and plastic table). This may potentially yield contamination of the Saharan signal in the dust collected. Most of the sample’s weight amounted to around 10 g (median value = 7.5 g), which provides sufficient material for conducting grain size, elementary composition by XRF, as well as ^137^Cs activities. Determined for most of the samples, these analyses allowed the selection of those samples considered as scientifically representative of long-range transported dust (*n* = 53 of 110; Supplementary Materials). Clay mineralogy and REE compositions as well as lead and plutonium isotope contents were measured for a selection of samples among those considered as scientifically representative. All analytical results presented in the article and the Supplementary Materials are available via the following open access link: https://zenodo.org/records/14003603.

### Grain size

Grain size distribution of all samples except one for which not enough material was available was measured at Laboratoire des Sciences du Climat et de l’Environnement (LSCE) (Gif-Sur-Yvette, France) in deionized agitated water, on the bulk powder, by laser diffraction using a Malvern Panalytical Mastersizer 3000, providing particle number concentrations between 0.01 and 3500 μm in diameter through 100 channels of same width in log scale. The measurement has been repeated five times for each sample, and the measurement with the lowest uncertainties (here presented with the weighted residuals) has been taken into account.

### X-ray fluorescence

Geochemical elementary composition was measured on 107 bulk samples by XRF with a nondestructive PANalytical Epsilon 3XLE spectrometer (LSCE, Gif sur Yvette, France). About 1 g of crushed sample was transferred into 10-mm-diameter cells for measurements. The LSCE calibration defined for these cells use 37 certified sediment standards to calibrate 18 elements (Mg, Al, Si, K, Ca, Ti, Cr, Mn, Fe, Co, Ni, Cu, Zn, Rb, Sr, Zr, Ba, and Pb). Concentrations were measured at 5 kV (tube current of 950 μA, without filter, under helium flow), with a counting time of 100 s. Each sample was analyzed twice, and each standard reference material [NCS DC 73306 and NCS DC 73309 (carbonate rocks) and JMs-1 (marine sediment)] was analyzed six times during the sample analysis sessions to check the stability exactitude and precision of the analyses. The recovery rate of an element in a geochemical standard has been calculated as measured values divided by the certified values. The median values (*n* = 18) of the recovery rate for each element were 97% for Mg, 91% for Al, 87% for Si, 96 for K, 100% for Ca, 99% for Ti, 110% for Cr, 96% for Mn, 99% for Fe, 159% for Co, 142% for Ni, 87% for Cu, 83% for Zn, 104% for Rb, 105% for Sr, 106% for Zr, 61% for Ba, and 92% for Pb. We applied the error propagation law (*57*) to calculate the reported uncertainties.

### TQ-ICP–MS for elemental and lead isotope analysis

Multielementary and lead isotopic analyses were applied to a selection of 17 of the scientifically representative samples distributed over all Western Europe latitudes affected by the dust event. The samples were sieved to 63 μm before digestion, the protocol of which is detailed in (*58*). Approximately 100 mg of the fine dust fraction was digested by successive additions of HF (4 ml, 47 to 51%) + HClO_4_ (2 ml, 65 to 71%) and then HCl (3.75 ml, 34 to 37%) + HNO_3_ (67%, 1.25 ml) (TraceMetal Grade, Thermo Fisher Scientific) in closed Teflon vessel on hot plates (DigiPREP, SCP Science). A certified reference material (IAEA lake sediment SL1) and a chemical blank were digested in the same way as the powder samples to control the mineralization efficiency and evaluate the potential contamination during digestion and subsequent analysis. The concentrations of Na, Mg, Al, K, Ca, Sc, Ti, V, Cr, Mn, Fe, Co, Ni, Cu, Zn, As, Se, Rb, Sr, Y, Mo, Ag, Cd, Sb, Cs, Ba, REEs, Tl, Pb, Th, and U were analyzed, as well as Pb radiogenic isotopes (204, 206, 207, and 208). Analyses were conducted using Triple Quadrupole Inductively Coupled Plasma Mass Spectrometry (TQ-ICP-MS) (Thermo Fisher Scientific iCAP) (*58*) . The concentration values obtained for Certified Reference Material (CRM) SL-1 remained within 15% of the certified (or informative) values except for As (82%, recovery rate in percent), Mo (138%), La (123%), and Ce (119%). The recovery rates for other elements were as follows: Na (98%), Mg (111%), K (102%), Sc (103%), Ti (86%), V (110%), Cr (100%), Mn (111%), Fe (107%), Co (91%), Ni (99%), Cu (95%), Zn (90%), Rb (96%), Sr (99%), Y (98%), Ag (96%), Cd (94%), Sb (97%), Cs (101%), Ba (114%), Pr (104%), Nd (98%), Sm (108%), Eu (103%), Gd (104%), Tb (101%), Dy (102%), Ho (96%), Er (97%), Tm (94%), Yb (96%), Lu (96%), Tl (106%), Pb (96%), Th (96%), and U (91%). The average quantity found in blank digestion remained under the detection limits for Sc, Co, Ag, Cs, Tb, Ho, Tl, and U. Blank correction was therefore not applied to these elements.

Pb isotope ratios (^206^Pb/^204^Pb, ^208^Pb/^204^Pb, ^206^Pb/^207^Pb, and ^208^Pb/^207^Pb) were determined for the same solutions as those used for elemental content determination, following the same procedures as those described in (*58*). Isotope ratios were thus corrected with repeated measurements of the Pb reference material NIST SRM-981 analyzed between every two samples. ^200^Hg has been measured, and ^204^Hg signal intensity has been calculated for ^204^Pb interference correction. The Pb in digestion blanks were around 0.005 ng ml^−1^, while the analyzed solutions were at 3 ng ml^−1^ after dilution of the digestion solution. The blank correction was therefore not conducted. Two IAEA Lake sediment SL-1 were analyzed, and the measured value was divided by certified values amounted to 100.23 and 100.33% for ^206^Pb/^204^Pb, 100.22 and 100.58% for ^208^Pb/^204^Pb, 100.35 and 100.33% for ^206^Pb/^207^Pb, and 100.33 and 100.58% for ^208^Pb/^207^Pb.

### Elemental EFs and compositional biplot

EF of each element (X) with respect to UCC was calculated as

EF(X)=[X]sample[Al]sample[X]UCC[Al]UCC (1)

where [*X*]_sample_ and [Al]_sample_ are concentrations of an element X and Al in samples and [*X*]_UCC_ and [Al]_UCC_ are average concentrations of X and Al in the upper crust (*27*).

For compositional data, the centered log-ratio (clr) transformation was calculated before applying multivariate techniques based on the following equation

$$\text{clr}\left(x\right)=\left[\text{ln}\frac{x_{1}}{\text{gm}\left(x\right)},\ldots ,\text{ln}\frac{x_{D}}{\text{gm}\left(x\right)}\right]$$

where *D* is the number of elements and gm(*x*) is the geometric mean of the *D* parts. Principal components analysis was then computed on the XRF and TQ-ICP–MS clr transformed data, with the “acomp” (closure operation) and “princomp” (principal components analysis projection) functions provided by the “compositions” package for the R software (*59*), which has been specifically designed to analyze compositional data (*60*).

### Clay mineralogy

A total of 15 samples were selected within the scientifically representative dataset because of their higher amount of material available and with the objective to cover the entire latitudinal transect of the dust event over Western Europe. Samples were sieved at 63 μm and treated with acetic acid (C_2_H_4_O_2_) buffered at 4.5 pH with sodium acetate for 24 to 48 hours (*61*) to remove potential calcium carbonates. Then, samples were leached with hydrogen peroxide (H_2_O_2_; 5%) for 24 hours to remove the organic matter. The clay size fraction (<2 to 4 μm) was isolated by centrifugation following the procedure described in (*62*). The clay mineralogy composition was carried out by x-ray diffraction (XRD) using a PANalytical diffractometer at the Geoscience Paris-Saclay laboratory (GEOPS, Paris-Saclay University, France) on oriented mounts. Three XRD determinations were performed: (i) on untreated samples; (ii) on glycolated samples (after saturation for 12 hours in ethylene glycol); and (iii) on samples heated at 490°C for 2 hours. Semiquantitative estimation of clay mineral abundances was performed using the Macintosh MacDiff 4.2.6 software (*63*). The analytical quality and the reproducibility of XRD measurements were controlled with replicated measurements, and an uncertainty of 5% was estimated.

### Radionuclides

The activities of γ-emitting radionuclides were analyzed in 107 samples with high-purity germanium detectors. Three samples were not analyzed because of an insufficient quantity of material available (<3 g) to obtain a detectable signal. The samples were weighed and sealed airtight in analysis containers, and most of them were analyzed at LSCE in Gif-sur-Yvette (France). Samples with insufficient material (<10 g) were sent to the Laboratoire Souterrain de Modane (France), where two well-type detectors are available to analyze a few grams of material in a lab devoid of cosmic background (*64*). In all samples, ^137^Cs was detected by its characteristic emission peak at 662 keV. Analyses lasted for ~80,000 s. Detector calibration and quality insurance were conducted using certified materials distributed by the IAEA.

A total of six samples were selected within the scientifically representative dataset and were analyzed for plutonium (Pu) isotope contents at Spiez laboratory (Switzerland) following procedures as published elsewhere (*65*–*67*). In summary, up to 5 g of aliquots of ashed material was used for radiochemical analysis. For determining the Pu isotope activities, a known amount of ^242^Pu (3 pg) was added as a radiochemical recovery tracer. The samples were digested by borate fusion, and the melt was dissolved in 4.5 M HNO_3_. After filtration of the silicates, Pu radionuclides were separated and purified using a TEVA extraction chromatography resin as described elsewhere (*68*). Mass spectrometric measurements were performed in a sector field ICP-MS (Element XR, Thermo Fisher Scientific). For the analysis of plutonium isotopes, the corresponding eluates were introduced into the ICP-MS Element XR with an Apex nebulizing system connected to an Advanced Control Module (ACM) desolvator (Elemental Scientific Incorporation) to enhance the signals. The interferences from uranium were corrected with tailing factors for the abundance sensitivity.

### Back trajectories and PM_10_ concentrations

Airmass backward trajectories were calculated using the Hybrid Single-Particle Lagrangian Integrated Trajectory (Hysplit) rev. 5.2 model (*69*) based on the Global Data Assimilation System for three sampling sites from South of Spain to Central France: Almeria, 36.50°N, 2.27°W; Alceda, 43.11°N, 3.55°W; and Sigloy, 47.83°N, 2.22°E. The backward trajectory length was 6 days long between the 15th and 18th of March 2022, and an ensemble calculation was initiated every 3 hours for each sampling site using code provided described in the “openair” R package (*70*). An arriving height at 3000 m was chosen on the basis of previous studies showing that the Saharan dust is mainly transported over the Western Mediterranean basin between 1.5 and 5 km in altitude (*71*) and that the dust was mainly transported between 1.5 and 6 km in altitude during the mid-March 2022 event (*21*). The backward trajectory output data were combined with available surface PM_10_ concentration time series data and visualized using QGIS software. Measured PM_10_ concentration data were imported from publicly accessible repositories that can be found at the following locations: (i) https://eea.europa.eu/en/datahub/datahubitem-view/3b390c9c-f321-490a-b25a-ae93b2ed80c1; (ii) https://discomap.eea.europa.eu/map/fme/AirQualityExport.htm. The web server space has been provided by Ricardo Energy & Environment.

### CAMS reanalysis

PM_10_ surface concentration reanalysis data were taken from the CAMS reanalysis, which is the latest global reanalysis dataset of atmospheric composition produced by the European Centre for Medium-Range Weather Forecasts (ECMWF) (*41*). Data were downloaded from https://ads.atmosphere.copernicus.eu/cdsapp#!/dataset/cams-global-reanalysis-eac4?tab=form (last accessed: 25 January 2024).

### North African Sandstorm Survey

The North African Sandstorm Survey (NASCube) provides remote sensing images to detect sandstorms over the Sahara and Saudi Arabia and estimate day and night aerosol optical depth (*40*). Animation and figures were downloaded from http://nascube.univ-lille1.fr.

## Supplementary Materials

### This PDF file includes:

Supplementary Text Figs. S1 to S3 References
