# Supplementary material for: Radioactive contamination transported to Western Europe with Saharan dust
Source: Sci Adv. 2025 Jan 31;11(5):eadr9192. doi: 10.1126/sciadv.adr9192 (PMC11784833; doi:10.1126/sciadv.adr9192)
Supplement: Supplementary file 1 — Supplementary Text Figs. S1 to S3 References [file sciadv.adr9192_sm.pdf]

Science Advances
AAAS

Supplementary Materials for

Radioactive contamination transported to Western Europe with Saharan dust

Yangjunjie Xu-Yang *et al.*

Corresponding author: Yangjunjie Xu-Yang, yangjunjie.xu@lsce.ipsl.fr

Sci. Adv. **11**, eadr9192 (2025)
DOI: 10.1126/sciadv.adr9192

This PDF file includes:

Supplementary Text
Figs. S1 to S3
References

Selection of the scientifically representative samples of the citizen science campaign

The active participation of the public allowed us to gather 110 samples (Figure S1a) collected in six different European countries including France (14 samples), Austria (12 samples), Spain (80 samples) and Belgium, Luxembourg and Germany (1 sample in each country). In March 2022, two successive Saharan dust events reached Western Europe. The beginning of the first and major one was detected from the 15th and yielded important atmospheric concentrations until the 18th, then progressively decreasing (*72*). The second outbreak started the 23nd. In this study, we focused on the first dust outbreak, during which most of the samples were collected. Consequently, samples with late collection date (later than 22/03/2022) were eliminated from the original data set (n=18). For the samples collected between the 15th and 22st of March, the variability in the types of exposed surfaces (e.g., metal roof and plastic tables etc.) and the delay between Saharan dust deposition and sample collection, may have favored contamination of the Saharan dust collected with local particles, as suggested by the presence of a significant mode of particles above 100 µm in diameter in the size distribution of many samples (Figure S2A). The coarse fraction of the size distribution of Saharan dust particles deposited in Western Europe is not precisely known due to the lack of direct measurements reported in the literature but we can refer to studies of European loess soils accumulated during glacial periods of high dust transport. The loess grain size distribution commonly present a major mode in the silty-clay size range (i.e. <63 µm in diameter) representing up to 80% of the total particle volume (*73*, *74*). To limit potential contamination by local coarse particles, only samples with 90% of their particle diameters (Dx(90)) smaller than 85 µm were selected. As a consequence, 35 samples were eliminated from the original data set. One additional sample could not be analyzed for its size distribution because of too small quantity and was consequently also removed from the data set. Interestingly, all the selected samples (n=56) present

a major particle size mode within the silt fraction (defined here by the proportion <66.9 μm corresponding to the channel with the limit closest to the standard 63 μm) >88%. Among these 56 samples, two other samples presenting an atypical grainsize distribution (Figure S2) suggesting a mixture with particles other than Saharan dust were also eliminated. Finally, one remaining sample presented anthropogenic Pb isotopes signature (*30*, *33–36*) and high concentration of Pb, was also discarded. Finally, 53 out of the 110 samples collected by the participative citizen science call were considered as scientifically representative of long-range transported Saharan dust. Most of them from Spain and France (Figure S1B). It is interesting to note that the relation between the modal diameter of the particle size distribution and latitude is clear, with a progressive transition to finer particle sizes from lower to higher latitude regions due to higher gravitational settling velocities of coarse particles (Figure S2B). Interestingly mineralogical and geochemical signatures obtained on both the fine (clay; mineralogy) or coarser (<63 μm; REE and Pb isotopes) particles fractions of some of the selected samples are systematically very similar, suggesting same provenance and limited contamination (Figure 2). Additionally, Pb isotopic signatures and elemental composition obtained are typically "natural" (*30*, *33–36*) (Figure 2), confirming the robustness of the selection.

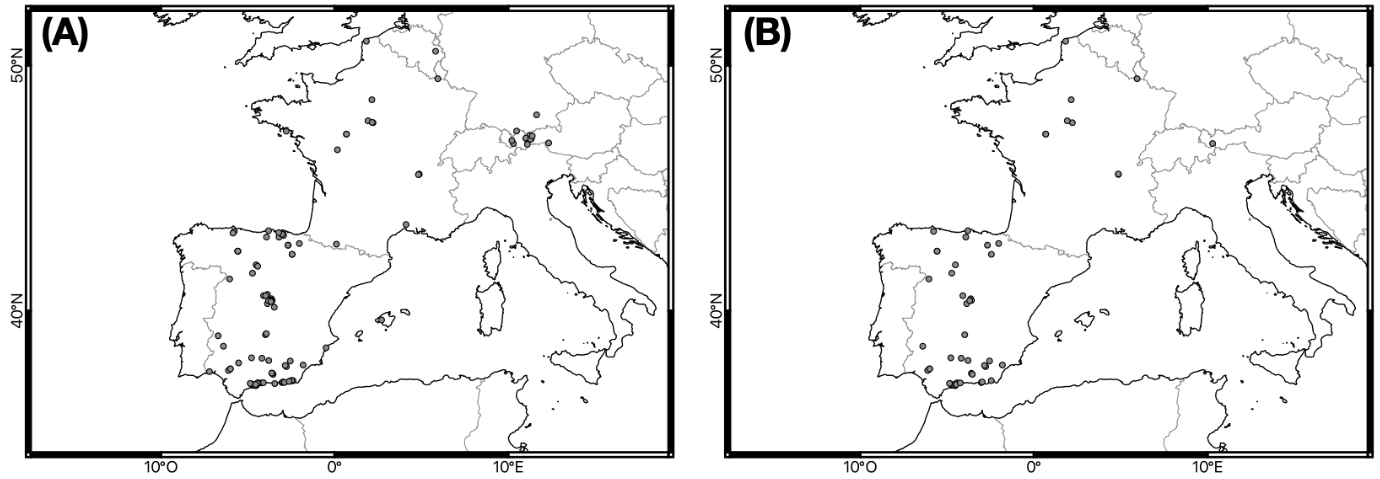

Fig. S1. Maps of locations sampled during the citizen science campaign. (A) Location of the 110 collected samples (B) Location of the 53 samples considered as scientifically representative of long-range transported dust based on the date of collection and grainsize distribution.

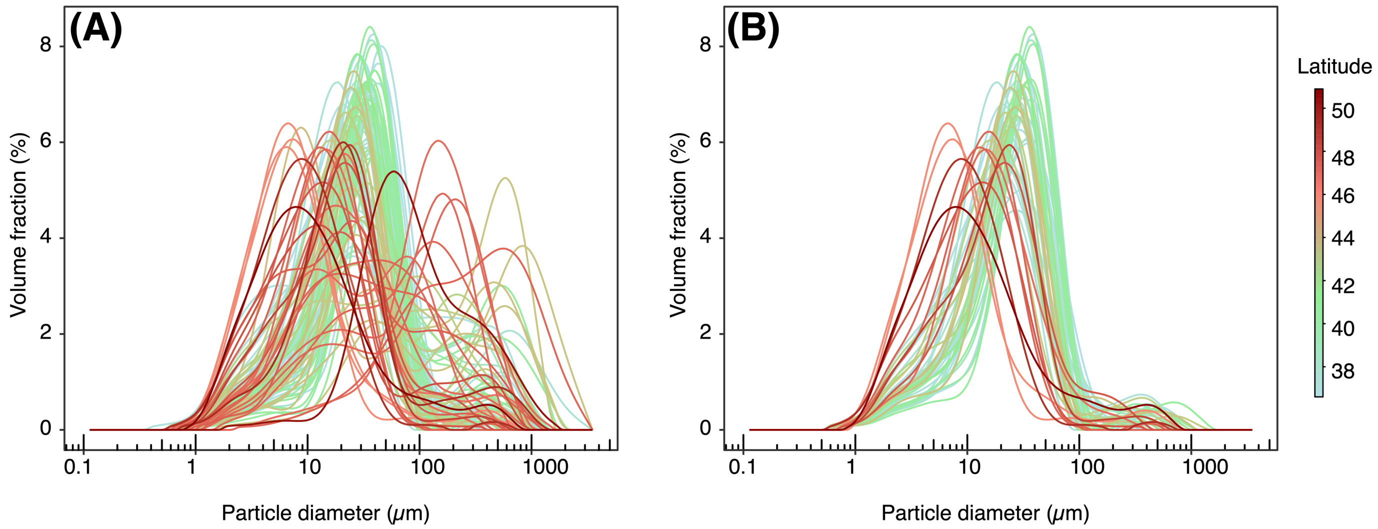

Fig. S2. Grainsize distribution of the dust deposition samples collected by citizens during the March 2022 Saharan dust event. (A) Distribution of 109 out of the 110 collected samples for which enough material was available for analyses; (B) Distribution of the 53 samples considered as scientifically representative.

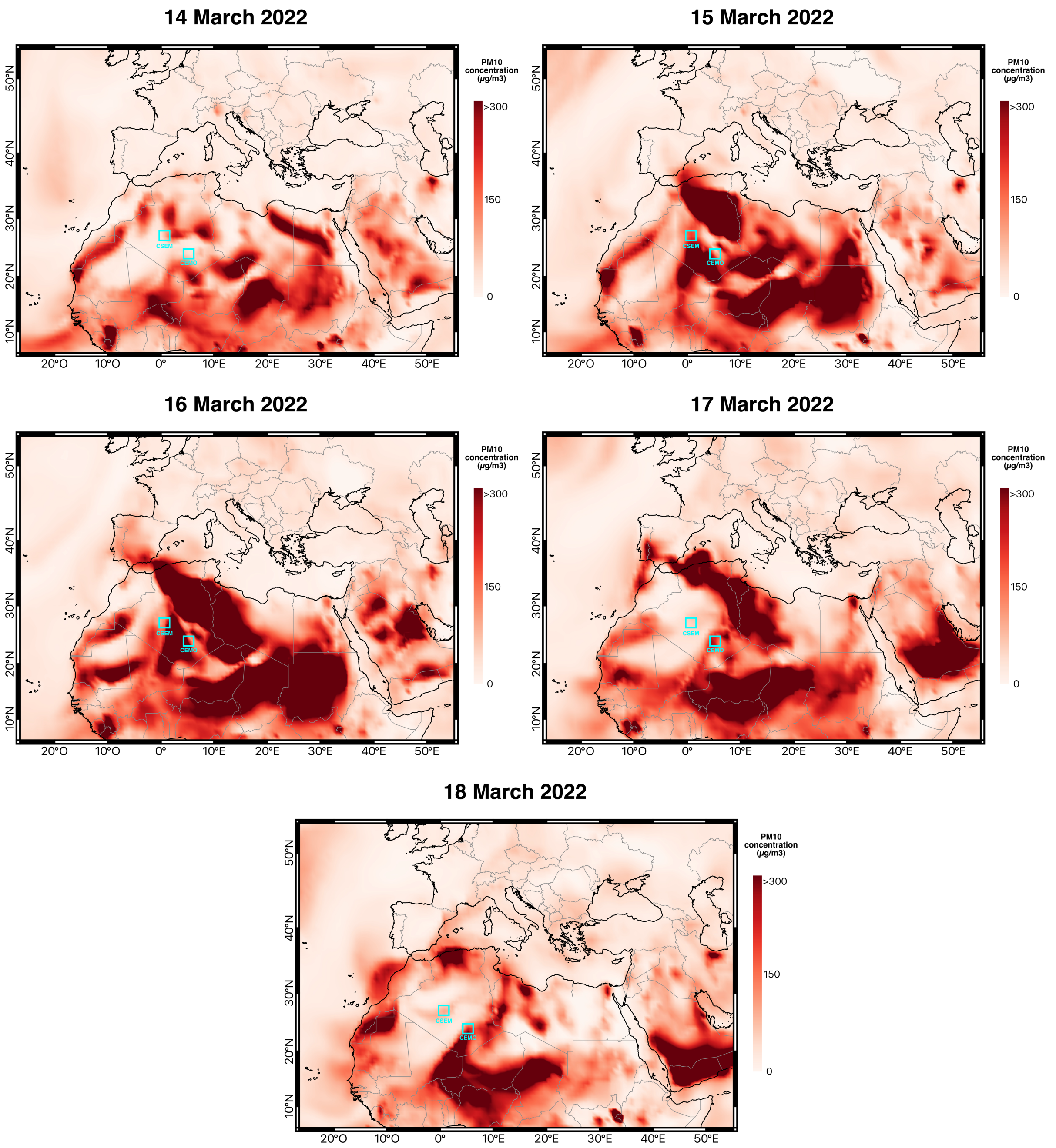

Fig. S3. Map of the March 2022 Saharan dust event PM10 concentration. The different panels represent the daily average surface PM_{10} concentration ($\mu g.m^{-3}$) for the 14-18 March 2022 temporal window derived from CAMS reanalysis of atmospheric composition ***(*****41*****)***.

REFERENCES AND NOTES

1. B. A. Maher, J. M. Prospero, D. Mackie, D. Gaiero, P. P. Hesse, Y. Balkanski, Global connections between aeolian dust, climate and ocean biogeochemistry at the present day and at the last glacial maximum. *Earth-Sci. Rev.* **99**, 61–97 (2010).

2. N. Mahowald, S. Albani, J. F. Kok, S. Engelstaeder, R. Scanza, D. S. Ward, M. G. Flanner, The size distribution of desert dust aerosols and its impact on the Earth system. *Aeolian Res.* **15**, 53–71 (2014).

3. N. Mahowald, D. S. Ward, S. Kloster, M. G. Flanner, C. L. Heald, N. G. Heavens, P. G. Hess, J.-F. Lamarque, P. Y. Chuang, Aerosol impacts on climate and biogeochemistry. *Annu. Rev. Env. Resour.* **36**, 45–74 (2011).

4. M. J. Kennedy, O. A. Chadwick, P. M. Vitousek, L. A. Derry, D. M. Hendricks, Changing sources of base cations during ecosystem development, Hawaiian Islands. *Geology* **26**, 1015–1018 (1998).

5. K. Pabortsava, R. S. Lampitt, J. Benson, C. Crowe, R. McLachlan, F. A. C. Le Moigne, C. Mark Moore, C. Pebody, P. Provost, A. P. Rees, G. H. Tilstone, E. M. S. Woodward, Carbon sequestration in the deep Atlantic enhanced by Saharan dust. *Nat. Geosci.* **10**, 189–194 (2017).

6. Q. Wang, J. Gu, X. Wang, The impact of Sahara dust on air quality and public health in European countries. *Atmos. Environ.* **241**, 117771 (2020).

7. X. Querol, A. Tobías, N. Pérez, A. Karanasiou, F. Amato, M. Stafoggia, C. Pérez García-Pando, P. Ginoux, F. Forastiere, S. Gumy, P. Mudu, A. Alastuey, Monitoring the impact of desert dust outbreaks for air quality for health studies. *Environ. Int.* **130**, 104867 (2019).

8. P. Ginoux, J. M. Prospero, T. E. Gill, N. C. Hsu, M. Zhao, Global-scale attribution of anthropogenic and natural dust sources and their emission rates based on MODIS Deep Blue aerosol products. *Rev. Geophys.* **50**, 10.1029/2012RG000388 (2012).

9. N. Huneeus, M. Schulz, Y. Balkanski, J. Griesfeller, J. Prospero, S. Kinne, S. Bauer, O. Boucher, M. Chin, F. Dentener, T. Diehl, R. Easter, D. Fillmore, S. Ghan, P. Ginoux, A. Grini,

L. Horowitz, D. Koch, M. C. Krol, W. Landing, X. Liu, N. Mahowald, R. Miller, J.-J. Morcrette, G. Myhre, J. Penner, J. Perlwitz, P. Stier, T. Takemura, C. S. Zender, Global dust model intercomparison in AeroCom phase I. *Atmos. Chem. Phys.* **11**, 7781–7816 (2011).

10. G. A. d'Almeida, A model for Saharan dust transport. *J. Appl. Meteorol. Climatol.* **25**, 903–916 (1986).

11. J. Pey, J. C. Larrasoaña, N. Pérez, J. C. Cerro, S. Castillo, M. L. Tobar, A. De Vergara, I. Vázquez, J. Reyes, M. P. Mata, T. Mochales, J. M. Orellana, J. Causapé, Phenomenology and geographical gradients of atmospheric deposition in southwestern Europe: Results from a multi-site monitoring network. *Sci. Total Environ.* **744**, 140745 (2020).

12. D. Francis, R. Fonseca, N. Nelli, D. Bozkurt, G. Picard, B. Guan, Atmospheric rivers drive exceptional Saharan dust transport towards Europe. *Atmos. Res.* **266**, 105959 (2022).

13. J. Vincent, B. Laurent, R. Losno, E. Bon Nguyen, P. Roullet, S. Sauvage, S. Chevaillier, P. Coddeville, N. Ouboulmane, A. G. Di Sarra, A. Tovar-Sánchez, D. Sferlazzo, A. Massanet, S. Triquet, R. Morales Baquero, M. Fornier, C. Coursier, K. Desboeufs, F. Dulac, G. Bergametti, Variability of mineral dust deposition in the western Mediterranean basin and south-east of France. *Atmos. Chem. Phys.* **16**, 8749–8766 (2016).

14. L. Matassoni, G. Pratesi, D. Centioli, F. Cadoni, F. Lucarelli, S. Nava, P. Malesani, Saharan dust contribution to PM10, PM2.5 and PM1 in urban and suburban areas of Rome: A comparison between single-particle SEM-EDS analysis and whole-sample PIXE analysis. *J. Environ. Monit.* **13**, 732 (2011).

15. O. Masson, D. Piga, R. Gurriaran, D. D'Amico, Impact of an exceptional Saharan dust outbreak in France: PM10 and artificial radionuclides concentrations in air and in dust deposit. *Atmos. Environ.* **44**, 2478–2486 (2010).

16. L. Menut, O. Masson, B. Bessagnet, Contribution of Saharan dust on radionuclide aerosol activity levels in Europe? The 21–22 February 2004 case study. *J. Geophys. Res.* **114**, 2009JD011767 (2009).

17. C. Papastefanou, M. Manolopoulou, S. Stoulos, A. Ioannidou, E. Gerasopoulos, Coloured rain dust from Sahara Desert is still radioactive. *J. Environ. Radioact.* **55**, 109–112 (2001).

18. R. Panchasi, "You don't screw with the Sahara": Radioactive Dust and the Return of the French Imperial Repressed (2023).

19. R. Cereceda, Irony as Saharan dust returns radiation from French nuclear tests in the 1960s, *Euronews* (2021). https://euronews.com/2021/03/01/irony-as-saharan-dust-returns-radiation-from-french-nuclear-tests-in-the-1960s.

20. P.-M. Puaud, Les sables du Sahara tombés sur nos voitures étaient radioactifs (2022). [Sahara sand that fell on our cars is radioactive.] https://france3-regions.francetvinfo.fr/normandie/calvados/caen/les-sables-du-sahara-tombes-sur-nos-voitures-sont-sans-doute-pollues-par-des-particules-radioactives-2503469.html.

21. E. Cuevas-Agulló, D. Barriopedro, R. D. García, S. Alonso-Pérez, J. J. González-Alemán, E. Werner, D. Suárez, J. J. Bustos, G. García-Castrillo, O. García, Á. Barreto, S. Basart, "Sharp increase of Saharan dust intrusions over the Western Mediterranean and Euro-Atlantic region in winters 2020–2022 and associated atmospheric circulation" [preprint, Aerosols/Atmospheric Modelling and Data Analysis/Troposphere/Physics (physical properties and processes), 2023]; https://doi.org/10.5194/egusphere-2023-1749.

22. J. Gomes, H. Esteves, L. Rente, Influence of an extreme Saharan dust event on the air quality of the West Region of Portugal. *Gases* **2**, 74–84 (2022).

23. G. Varga, O. Meinander, Á. Rostási, P. Dagsson-Waldhauserova, A. Csávics, F. Gresina, Saharan, Aral-Caspian and Middle East dust travels to Finland (1980–2022). *Environ. Int.* **180**, 108243 (2023).

24. IAEA, "Radiological Conditions at the Former French Nuclear Test Sites in Algeria: Preliminary Assessment and Recommendations" (STI/PUB/1215, 2005); https://iaea.org/publications/7174/radiological-conditions-at-the-former-french-nuclear-test-sites-in-algeria-preliminary-assessment-and-recommendations.

25. United Nations, Ed., *Sources and Effects of Ionizing Radiation: United Nations Scientific Committee on the Effects of Atomic Radiation: UNSCEAR 2000 Report to the General Assembly, with Scientific Annexes* (United Nations, 2000).

26. O. Evrard, Call for participation of citizen science for the study of radioactive contamination transported to Western Europe with Saharan dust (2022). https://x.com/EvrardOlivier/status/1504342715932282883.

27. R. L. Rudnick, S. Gao, "Composition of the continental crust" in *Treatise on Geochemistry* (Elsevier, 2014; https://linkinghub.elsevier.com/retrieve/pii/B9780080959757003016), pp. 1–51.

28. C. R. Lawrence, J. C. Neff, The contemporary physical and chemical flux of aeolian dust: A synthesis of direct measurements of dust deposition. *Chem. Geol.* **267**, 46–63 (2009).

29. A. Avila, I. Queralt-Mitjans, M. Alarcón, Mineralogical composition of African dust delivered by red rains over northeastern Spain. *J. Geophys. Res.* **102**, 21977–21996 (1997).

30. D. Guinoiseau, S. P. Singh, S. J. G. Galer, W. Abouchami, R. Bhattacharyya, K. Kandler, C. Bristow, M. O. Andreae, Characterization of Saharan and Sahelian dust sources based on geochemical and radiogenic isotope signatures. *Quat. Sci. Rev.* **293**, 107729 (2022).

31. D. Scheuvens, L. Schütz, K. Kandler, M. Ebert, S. Weinbruch, Bulk composition of northern African dust and its source sediments — A compilation. *Earth Sci. Rev.* **116**, 170–194 (2013).

32. J. M. Prospero, Environmental characterization of global sources of atmospheric soil dust identified with the NIMBUS 7 Total Ozone Mapping Spectrometer (TOMS) absorbing aerosol product. *Rev. Geophys.* **40**, 10.1029/2000RG000095 (2002).

33. A. Bollhöfer, W. Chisholm, K. J. R. Rosman, Sampling aerosols for lead isotopes on a global scale. *Anal. Chim. Acta* **390**, 227–235 (1999).

34. A. Bollhöfer, K. J. R. Rosman, Isotopic source signatures for atmospheric lead: The Northern Hemisphere. *Geochim. Cosmochim. Acta* **65**, 1727–1740 (2001).

35. A. Bollhöfer, K. J. R. Rosman, Isotopic source signatures for atmospheric lead: The Southern Hemisphere. *Geochim. Cosmochim. Acta* **64**, 3251–3262 (2000).

36. D. Widory, S. Roy, Y. Le Moullec, G. Goupil, A. Cocherie, C. Guerrot, The origin of atmospheric particles in Paris: A view through carbon and lead isotopes. *Atmos. Environ.* **38**, 953–961 (2004).

37. S. Engelstaedter, I. Tegen, R. Washington, North African dust emissions and transport. *Earth Sci. Rev.* **79**, 73–100 (2006).

38. A. T. Evan, S. Fiedler, C. Zhao, L. Menut, K. Schepanski, C. Flamant, O. Doherty, Derivation of an observation-based map of North African dust emission. *Aeolian Res.* **16**, 153–162 (2015).

39. E. Molinaroli, "Mineralogical characterisation of Saharan dust with a view to its final destination in Mediterranean sediments" in *The Impact of Desert Dust Across the Mediterranean*, S. Guerzoni, R. Chester, Eds. (Springer Netherlands, 1996; http://link.springer.com/10.1007/978-94-017-3354-0_14), vol. 11 of *Environmental Science and Technology Library*, pp. 153–162.

40. L. Gonzalez, X. Briottet, North Africa and Saudi Arabia Day/Night Sandstorm Survey (NASCube). *Remote Sens.* **9**, 896 (2017).

41. A. Inness, M. Ades, A. Agustí-Panareda, J. Barré, A. Benedictow, A.-M. Blechschmidt, J. J. Dominguez, R. Engelen, H. Eskes, J. Flemming, V. Huijnen, L. Jones, Z. Kipling, S. Massart, M. Parrington, V.-H. Peuch, M. Razinger, S. Remy, M. Schulz, M. Suttie, The CAMS reanalysis of atmospheric composition. *Atmos. Chem. Phys.* **19**, 3515–3556 (2019).

42. K. O. Buesseler, E. R. Sholkovitz, The geochemistry of fallout plutonium in the North Atlantic: II. $^{240}Pu^{239}Pu$ ratios and their significance. *Geochim. Cosmochim. Acta* **51**, 2623–2637 (1987).

43. P. W. Krey, E. P. Hardy, C. Pachucki, F. Rourke, J. Coluzza, W. K. Benson, *Mass Isotopic Composition of Global Fall-out Plutonium in Soil* [IAEA, International Atomic Energy Agency (IAEA), 1976; http://inis.iaea.org/search/search.aspx?orig_q=RN:08332318].

44. K. Meusburger, O. Evrard, C. Alewell, P. Borrelli, G. Cinelli, M. Ketterer, L. Mabit, P. Panagos, K. Van Oost, C. Ballabio, Plutonium aided reconstruction of caesium atmospheric fallout in European topsoils. *Sci. Rep.* **10**, 11858 (2020).

45. R. García-Tenorio, ^{240}Pu/^{239}Pu atom ratio as a fingerprint of local and tropospheric fallout due to events involving nuclear weapons: A review. *J. Rad. Nucl. Appl* **3**, 65–77 (2018).

46. V. Valković, *Radioactivity in the Environment* (Elsevier Science B.V., 1st ed., 2000).

47. M. Koide, K. K. Bertine, T. J. Chow, E. D. Goldberg, The240Pu239Pu ratio, a potential geochronometer. *Earth Planet. Sci. Lett.* **72**, 1–8 (1985).

48. M. Dumont, S. Gascoin, M. Réveillet, D. Voisin, F. Tuzet, L. Arnaud, M. Bonnefoy, M. Bacardit Peñarroya, C. Carmagnola, A. Deguine, A. Diacre, L. Dürr, O. Evrard, F. Fontaine, A. Frankl, M. Fructus, L. Gandois, I. Gouttevin, A. Gherab, P. Hagenmuller, S. Hansson, H. Herbin, B. Josse, B. Jourdain, I. Lefevre, G. Le Roux, Q. Libois, L. Liger, S. Morin, D. Petitprez, A. Robledano, M. Schneebeli, P. Salze, D. Six, E. Thibert, J. Trachsel, M. Vernay, L. Viallon-Galinier, C. Voiron, "Spatial variability of Saharan dust deposition revealed through a citizen science campaign" (preprint, ESSD–Atmosphere/Atmospheric chemistry and physics, 2023); https://doi.org/10.5194/essd-2023-16.

49. O. Evrard, P.-A. Chaboche, R. Ramon, A. Foucher, J. P. Laceby, A global review of sediment source fingerprinting research incorporating fallout radiocesium (137Cs). *Geomorphology* **362**, 107103 (2020).

50. M. K. Pham, E. Chamizo, J. L. Mas Balbuena, J.-C. Miquel, J. Martín, I. Osvath, P. P. Povinec, Impact of Saharan dust events on radionuclide levels in Monaco air and in the water column of the northwest Mediterranean Sea. *J. Environ. Radioact.* **166**, 2–9 (2017).

51. K. Meusburger, L. Mabit, M. Ketterer, J.-H. Park, T. Sandor, P. Porto, C. Alewell, A multi-radionuclide approach to evaluate the suitability of 239+240Pu as soil erosion tracer. *Sci. Total Environ.* **566-567**, 1489–1499 (2016).

52. Council of the European Union, *Council Regulation* (*Euratom*) *2016/52 of 15 January 2016 Laying down Maximum Permitted Levels of Radioactive Contamination of Food and Feed Following a Nuclear Accident or Any Other Case of Radiological Emergency*, *and Repealing Regulation* (*Euratom*) *No 3954/87 and Commission Regulations* (*Euratom*) *No 944/89 and* (*Euratom*) *No 770/90* (2016; http://data.europa.eu/eli/reg/2016/52/oj) vol. JO L 13 du 20.1.2016.

53. J. D. Pleil, M. A. G. Wallace, M. D. Davis, C. M. Matty, The physics of human breathing: Flow, timing, volume, and pressure parameters for normal, on-demand, and ventilator respiration. *J. Breath Res.* **15**, 10.1088/1752-7163/ac2589 (2021).

54. ICRP, K. Eckerman, J. Harrison, H.-G. Menzel, C. H. Clement, ICRP Publication 119: Compendium of Dose Coefficients Based on ICRP Publication 60. *Annals of the ICRP* **42**, 1-130 (2012).

55. Council of the European Union, *Council Directive 2013/59/Euratom of 5 December 2013 Laying down Basic Safety Standards for Protection against the Dangers Arising from Exposure to Ionising Radiation*, *and Repealing Directives 89/618/Euratom*, *90/641/Euratom*, *96/29/Euratom*, *97/43/Euratom and 2003/122/Euratom* (2013; http://data.europa.eu/eli/dir/2013/59/oj).

56. F. Colas, B. Zanda, S. Bouley, S. Jeanne, A. Malgoyre, M. Birlan, C. Blanpain, J. Gattacceca, L. Jorda, J. Lecubin, C. Marmo, J. L. Rault, J. Vaubaillon, P. Vernazza, C. Yohia, D. Gardiol, A. Nedelcu, B. Poppe, J. Rowe, M. Forcier, D. Koschny, J. M. Trigo-Rodriguez, H. Lamy, R. Behrend, L. Ferrière, D. Barghini, A. Buzzoni, A. Carbognani, M. Di Carlo, M. Di Martino, C. Knapic, E. Londero, G. Pratesi, S. Rasetti, W. Riva, G. M. Stirpe, G. B. Valsecchi, C. A. Volpicelli, S. Zorba, D. Coward, E. Drolshagen, G. Drolshagen, O. Hernandez, E. Jehin, M. Jobin, A. King, C. Nitschelm, T. Ott, A. Sanchez-Lavega, A. Toni, P. Abraham, F. Affaticati, M. Albani, A. Andreis, T. Andrieu, S. Anghel, E. Antaluca, K. Antier, T. Appéré, A. Armand, G. Ascione, Y. Audureau, G. Auxepaules, T. Avoscan, D. B. Aissa, P. Bacci, O. Bădescu, R.

Baldini, R. Baldo, A. Balestrero, D. Baratoux, E. Barbotin, M. Bardy, S. Basso, O. Bautista, L. D. Bayle, P. Beck, R. Bellitto, R. Belluso, C. Benna, M. Benammi, E. Beneteau, Z. Benkhaldoun, P. Bergamini, F. Bernardi, M. E. Bertaina, P. Bessin, L. Betti, F. Bettonvil, D. Bihel, C. Birnbaum, O. Blagoi, E. Blouri, I. Boacă, R. Boată, B. Bobiet, R. Bonino, K. Boros, E. Bouchet, V. Borgeot, E. Bouchez, D. Boust, V. Boudon, T. Bouman, P. Bourget, S. Brandenburg, P. Bramond, E. Braun, A. Bussi, P. Cacault, B. Caillier, A. Calegaro, J. Camargo, S. Caminade, A. P. C. Campana, P. Campbell-Burns, R. Canal-Domingo, O. Carell, S. Carreau, E. Cascone, C. Cattaneo, P. Cauhape, P. Cavier, S. Celestin, A. Cellino, M. Champenois, H. C. Aoudjehane, S. Chevrier, P. Cholvy, L. Chomier, A. Christou, D. Cricchio, P. Coadou, J. Y. Cocaign, F. Cochard, S. Cointin, E. Colombi, J. P. C. Saavedra, L. Corp, M. Costa, F. Costard, M. Cottier, P. Cournoyer, E. Coustal, G. Cremonese, O. Cristea, J. C. Cuzon, G. D'Agostino, K. Daiffallah, C. Dănescu, A. Dardon, T. Dasse, C. Davadan, V. Debs, J. P. Defaix, F. Deleflie, M. D'Elia, P. De Luca, P. De Maria, P. Deverchère, H. Devillepoix, A. Dias, A. Di Dato, R. Di Luca, F. M. Dominici, A. Drouard, J. L. Dumont, P. Dupouy, L. Duvignac, A. Egal, N. Erasmus, N. Esseiva, A. Ebel, B. Eisengarten, F. Federici, S. Feral, G. Ferrant, E. Ferreol, P. Finitzer, A. Foucault, P. Francois, M. Frîncu, J. L. Froger, F. Gaborit, V. Gagliarducci, J. Galard, A. Gardavot, M. Garmier, M. Garnung, B. Gautier, B. Gendre, D. Gerard, A. Gerardi, J. P. Godet, A. Grandchamps, B. Grouiez, S. Groult, D. Guidetti, G. Giuli, Y. Hello, X. Henry, G. Herbreteau, M. Herpin, P. Hewins, J. J. Hillairet, J. Horak, R. Hueso, E. Huet, S. Huet, F. Hyaumé, G. Interrante, Y. Isselin, Y. Jeangeorges, P. Janeux, P. Jeanneret, K. Jobse, S. Jouin, J. M. Jouvard, K. Joy, J. F. Julien, R. Kacerek, M. Kaire, M. Kempf, D. Koschny, C. Krier, M. K. Kwon, L. Lacassagne, D. Lachat, A. Lagain, E. Laisné, V. Lanchares, J. Laskar, M. Lazzarin, M. Leblanc, J. P. Lebreton, J. Lecomte, P. Le Dû, F. Lelong, S. Lera, J. F. Leoni, A. Le-Pichon, P. Le-Poupon, A. Leroy, G. Leto, A. Levansuu, E. Lewin, A. Lienard, D. Licchelli, H. Locatelli, S. Loehle, D. Loizeau, L. Luciani, M. Maignan, F. Manca, S. Mancuso, E. Mandon, N. Mangold, F. Mannucci, L. Maquet, D. Marant, Y. Marchal, J. L. Marin, J. C. Martin-Brisset, D. Martin, D. Mathieu, A. Maury, N. Mespoulet, F. Meyer, J. Y. Meyer, E. Meza, V. M. Cecchi, J. J. Moiroud, M. Millan, M. Montesarchio, A. Misiano, E. Molinari, S. Molau, J. Monari, B. Monflier, A. Monkos, M. Montemaggi, G. Monti, R. Moreau, J. Morin, R. Mourgues, O. Mousis, C. Nablanc, A. Nastasi, L. Niacşu, P. Notez, M. Ory, E. Pace, M. A. Paganelli, A. Pagola, M. Pajuelo, J. F. Palacián, G. Pallier, P. Paraschiv, R. Pardini, M. Pavone, G. Pavy, G. Payen, A.

Pegoraro, E. Peña-Asensio, L. Perez, S. Pérez-Hoyos, V. Perlerin, A. Peyrot, F. Peth, V. Pic, S. Pietronave, C. Pilger, M. Piquel, T. Pisanu, M. Poppe, L. Portois, J. F. Prezeau, N. Pugno, C. Quantin, G. Quitté, N. Rambaux, E. Ravier, U. Repetti, S. Ribas, C. Richard, D. Richard, M. Rigoni, J. P. Rivet, N. Rizzi, S. Rochain, J. F. Rojas, M. Romeo, M. Rotaru, M. Rotger, P. Rougier, P. Rousselot, J. Rousset, D. Rousseu, O. Rubiera, R. Rudawska, J. Rudelle, J. P. Ruguet, P. Russo, S. Sales, O. Sauzereau, F. Salvati, M. Schieffer, D. Schreiner, Y. Scribano, D. Selvestrel, R. Serra, L. Shengold, A. Shuttleworth, R. Smareglia, S. Sohy, M. Soldi, R. Stanga, A. Steinhausser, F. Strafella, S. S. Mbaye, A. R. D. Smedley, M. Tagger, P. Tanga, C. Taricco, J. P. Teng, J. O. Tercu, O. Thizy, J. P. Thomas, M. Tombelli, R. Trangosi, B. Tregon, P. Trivero, A. Tukkers, V. Turcu, G. Umbriaco, E. Unda-Sanzana, R. Vairetti, M. Valenzuela, G. Valente, G. Varennes, S. Vauclair, J. Vergne, M. Verlinden, M. Vidal-Alaiz, R. Vieira-Martins, A. Viel, D. C. Vîntdevară, V. Vinogradoff, P. Volpini, M. Wendling, P. Wilhelm, K. Wohlgemuth, P. Yanguas, R. Zagarella, A. Zollo, FRIPON: A worldwide network to track incoming meteoroids. *Astron. Astrophys.* **644**, A53 (2020).

57. H. H. Ku, Notes on the use of propagation of error formulas. *J. Res. Natl. Bur. Stand.* **70C**, 263 (1966).

58. J. Briard, S. Ayrault, M. Roy-Barman, L. Bordier, M. L'Héritier, A. Azéma, D. Syvilay, S. Baron, Determining the geochemical fingerprint of the lead fallout from the Notre-Dame de Paris fire: Lessons for a better discrimination of chemical signatures. *Sci. Total Environ.* **864**, 160676 (2023).

59. R Core Team, R: A Language and Environment for Statistical Computing, R Foundation for Statistical Computing (2021); https://R-project.org/.

60. K. G. van den Boogaart, R. Tolosana-Delgado, *Analyzing Compositional Data with R* (Springer Berlin Heidelberg, 2013; http://link.springer.com/10.1007/978-3-642-36809-7).

61. P. E. Biscaye, Mineralogy and sedimentation of recent deep-sea clay in the Atlantic Ocean and adjacent seas and oceans. *GSA Bull.* **76**, 803–832 (1965).

62. G. Bayon, S. Toucanne, C. Skonieczny, L. André, S. Bermell, S. Cheron, B. Dennielou, J. Etoubleau, N. Freslon, T. Gauchery, Y. Germain, S. J. Jorry, G. Ménot, L. Monin, E.

Ponzevera, M.-L. Rouget, K. Tachikawa, J. A. Barrat, Rare earth elements and neodymium isotopes in world river sediments revisited. *Geochim. Cosmochim. Acta* **170**, 17–38 (2015).

63. R. Petschick, *MacDiff 4.1. 2. Powder Diffraction Software* (2000; http://geol. unierlangen. de/html/software/Macdiff. html).

64. J. L. Reyss, S. Schmidt, F. Legeleux, P. Bonte, Large, low background well-type detectors for measurements of environmental radioactivity. *Nucl. Instrum. Methods Phys. Res., Sect. A* **357**, 391–397 (1995).

65. S. Röllin, H. Sahli, R. Holzer, M. Astner, M. Burger, PU and NP analysis of soil and sediment samples with ICP-MS. *Appl. Radiat. Isot.* **67**, 821–827 (2009).

66. S. Röllin, J. A. Corcho-Alvarado, H. Sahli, V. Putyrskaya, E. Klemt, High-resolution records of cesium, plutonium, americium, and uranium isotopes in sediment cores from Swiss lakes. *Environ. Sci. Pollut. Res.* **29**, 85777–85788 (2022).

67. H. Sahli, S. Röllin, V. Putyrskaya, E. Klemt, B. Balsiger, M. Burger, J. A. Corcho Alvarado, A procedure for the sequential determination of radionuclides in soil and sediment samples. *J. Radioanal. Nucl. Chem.* **314**, 2209–2218 (2017).

68. S. Röllin, H. Sahli, L. Gnägi, J. A. Corcho Alvarado, Determination of plutonium and uranium radionuclides in glacier ice samples by MC-ICP-MS. *Chimia* **74**, 989 (2022).

69. A. F. Stein, R. R. Draxler, G. D. Rolph, B. J. B. Stunder, M. D. Cohen, F. Ngan, NOAA's HYSPLIT atmospheric transport and dispersion modeling system. *Bull. Am. Meteorol. Soc.* **96**, 2059–2077 (2015).

70. D. C. Carslaw, K. Ropkins, *openair*—An R package for air quality data analysis. *Environ. Model. Softw.* **27–28**, 52–61 (2012).

71. A. Gkikas, S. Basart, N. Hatzianastassiou, E. Marinou, V. Amiridis, S. Kazadzis, J. Pey, X. Querol, O. Jorba, S. Gassó, J. M. Baldasano, Mediterranean intense desert dust outbreaks and their vertical structure based on remote sensing data. *Atmos. Chem. Phys.* **16**, 8609–8642 (2016).

72. E. Liger, F. Hernández, F. J. Expósito, J. P. Díaz, P. A. Salazar-Carballo, E. Gordo, C. González, M. López-Pérez, Transport and deposition of radionuclides from northern Africa to the southern Iberian Peninsula and the Canary Islands during the intense dust intrusions of March 2022. *Chemosphere* **352**, 141303 (2024).

73. D. Rousseau, C. Chauvel, A. Sima, C. Hatté, F. Lagroix, P. Antoine, Y. Balkanski, M. Fuchs, C. Mellett, M. Kageyama, G. Ramstein, A. Lang, European glacial dust deposits: Geochemical constraints on atmospheric dust cycle modeling. *Geophys. Res. Lett.* **41**, 7666–7674 (2014).

74. D.-D. Rousseau, E. Derbyshire, P. Antoine, C. Hatté, "European Loess Records" in *Reference Module in Earth Systems and Environmental Sciences* (Elsevier, 2018; https://linkinghub.elsevier.com/retrieve/pii/B9780124095489111364), p. B9780124095489111364.
